# Local and systemic humoral response to ovine mastitis caused by *Staphylococcus epidermidis*

**DOI:** 10.1177/2050312118801466

**Published:** 2018-09-24

**Authors:** Maria Cristina Queiroga

**Affiliations:** 1 Departamento de Medicina Veterinária, Universidade de Évora, Évora, Portugal; 2 Instituto de Ciências Agrárias e Ambientais Mediterrânicas (ICAAM), Universidade de Évora, Évora, Portugal

**Keywords:** Veterinary immunology, veterinary bacteriology, infectious diseases, mastitis, immunoglobulin

## Abstract

**Objectives:**

Mastitis is responsible for a decrease in milk yield and quality. Disease control is vital for producers’ profit and for consumer’s welfare. This study aimed at investigating the immune response to *Staphylococcus epidermidis* intramammary infection.

**Methods:**

A total of 14 *S. epidermidis* isolates from milk collected from ewes with subclinical mastitis were used. Protein extracts were prepared and analysed by sodium dodecyl sulphate–polyacrylamide gel electrophoresis. Immunoblotting assay was performed for the detection of specific IgG and IgA in blood and milk from *S. epidermidis* mastitic ewes and from healthy animals.

**Results:**

The presence of pathogen-specific IgG was detected in blood of both infected and healthy animals. However, in milk, pathogen-specific IgG was only identified in infected animals, while IgA was found in both groups. Proteins with 59 and 43 kDa were recognized by all immunoglobulins screened in blood and milk provided by both healthy and mastitic ewes. In addition, in milk, IgG and IgA for proteins with 35 kDa were also detected.

**Conclusion:**

The results have lead to propose a theory for immunoglobulin dynamics in mammary gland’s defence: blood IgG1, specifically targeting intestinal antigens, is transported to the mammary gland with the main purpose of protecting the newborn, while IgG2 is specific for mammary pathogens and is transported to the mammary gland exclusively during inflammation. This study suggests that only local immunization should trigger IgG-producing cells in the mammary gland as a response to mastitis antigens. Moreover, IgA seems to be of crucial value for the defence of the ewe mammary gland, and stimulation strategies towards an increase in IgA should be addressed for mastitis prevention.

## Introduction

Mastitis prevalence in dairy sheep flocks may be quite high, with subclinical mastitis figures, in Portugal, ranging from 1% to 92.5%, with an average prevalence of 32.2%^1^ Other countries refer ewes’ prevalence rates between 0% and 85%.^2–16^ These numbers justify the importance of implementing prophylactic measures such as hygienic procedures during milking routine, which are undoubtedly crucial to reduce microorganism access to the mammary gland. Furthermore, the drying off treatment with antimicrobials, currently used as prophylactic management of mastitis in cattle, has already proved to be effective in the control of mastitis in small ruminants.^17^ However, this practice exerts a selection pressure for resistant strains^18^ and should be avoided.^19^

The stimulation of the mammary gland defence mechanisms for mastitis prophylaxis and treatment may be an alternative to the use of antimicrobials, with obvious advantages for Public Health. Although experimental work showing the possible improvement of resistance to intramammary infection has been performed, no strategy was ever developed to provide desirable protection levels.^20–22^ A 70% protection for *Staphylococcus aureus* infection and complete protection from inflammatory reactions, proven by somatic cell counts, was probably the best outcome^23^ of a developed vaccine, but a field trial with *Staphylococcus chromogenes* natural infection showed that 13.5% (5/37) heifers of the immunized group were infected at parturition, compared to 42.9% (18/42) in the non-immunized group,^24^ representing merely a 29.4% reduction.

Phagocytic cells, such as macrophages and neutrophils, which destroy and eliminate invading agents, constitute the major immune sentinels of the mammary gland.^25^ The quicker and efficient is the clear up; the smaller will be the damage extent caused to the mammary epithelium and the sooner the complete remission.^26,27^ In milk, phagocytes are less effective than in serum due to the ingestion of fat globules and casein and to the reduction of energy reserves during diapedesis.^28,29^ Bacterial opsonization enhances phagocytosis and antibodies are known as the most efficient opsonins.^30^ Immunoglobulin G (IgG) is the main isotype in ruminants milk and IgG2 is considered to be the main opsonin supporting neutrophil phagocytosis in milk of infected mammary glands,^31^ as bovine neutrophils and macrophages have Fc receptors that specifically bind to IgG2.^30^

The immunology studies of dairy ruminant’s mammary gland have focused mainly on the innate immune response and little is known on the immunoglobulin’s role in the mammary gland defence mechanisms.^32^ Although previous work has assessed the immunoglobulin response to vaccines in serum and milk whey,^33–38^ they addressed mainly IgG, and much of the immunoglobulin dynamics in the mammary gland is still to be acknowledged. Contrasting with non-ruminant species, IgA is present in low quantities in ruminants’ mammary gland, although it has been recognized as an important mucosal antibody able to perform immune exclusion, a key defensive mechanism at mucosal surfaces.^30^

The study of sheep immune response to infection is essential to develop strategies to stimulate mammary gland defence mechanisms and to improve mastitis prophylaxis. The aim of this study was to evaluate mammary and systemic humoral immune response to immune-relevant antigens from *Staphylococcus epidermidis*, the main aetiological agent of sheep mastitis in Portugal.^1^

## Materials and methods

### Animals

This is an exploratory study to gather preliminary information with the objective to acquire new insights into the mechanisms of immune response in the mammary gland. In an exploratory study sample, sizes may be small. These studies generate qualitative information.

Five ewes with *S. epidermidis* intramammary infection (IMI) in one udder half, according to the National Mastitis Council methodology,^39^ the other udder half being culture-negative, and two ewes with both udder halves culture-negative were used to provide blood serum and milk whey. All ewes were at mid-lactation and without recognized prior mastitis history.

Blood was collected in Vacutainer^®^ tubes with sodium citrate, centrifuged at 2000 × g for 15 min and then filtered through a 0.20 μm membrane (Acrodisc 4192; Gelman) and frozen at −20°C in sterile microtubes. Milk was aseptically collected and centrifuged at 26,890 × g at 4°C for 1 h. The fat layer was removed and the supernatant was transferred to another tube and again centrifuged under the same conditions for 1 h. The obtained whey was serially filtered through membranes of size 5 μm (Acro 50A 4264; Gelman), 0.45 μm (Acro 50A 4262; Gelman) and 0.20 μm (Acro 50A 4260; Gelman) and frozen at −20°C in sterile microtubes.

Ethical approval for this study was waived by Animal Welfare Body (Animal Research Ethics Committee of the University of Évora (ORBEA-UÉ)), because the Directive 2010/63/EU of the European Parliament and of the Council of 22 September 2010 on the protection of animals used for scientific purposes does not apply since the milk and blood collection practices were undertaken for the purposes of recognized animal husbandry, are non-experimental clinical veterinary practices and not likely to cause pain, suffering, distress or lasting harm higher than those equivalent to that caused by the introduction of a needle in accordance with good veterinary practice (Chapter I, Article 1, no. 5 (a), (b) and (d) of the Directive 2010/63/EU).

### Bacterial isolates

In all, 14 *S. epidermidis* isolates from milk collected from ewes at mid-lactation, belonging to several flocks, with unilateral or bilateral subclinical intramammary infection caused exclusively by *S. epidermidis* were used. Milk samples were aseptically collected into a sterilized container, after the teat was disinfected with 70% ethanol and the first flush rejected. Samples were kept refrigerated until processed, always on the day of collection. Bacteriological analyses were processed according to the National Mastitis Council methodology.^39^ Isolates were identified as *S. epidermidis* by standard procedures, including Gram staining, catalase test, biochemical characterization, using API-Staph (bioMérieux), and by internally transcribed spacer-polymerase chain reaction (ITS-PCR).^40^ Bacteria were stored at −80ºC.

### Sodium dodecyl sulphate–polyacrylamide gel electrophoresis and western blot procedure

Bacteria were grown overnight in 25 mL brain heart infusion broth (BHIB; CM225; Oxoid) at 37°C. Following centrifugation at 10,000 × g for 15 min at 4ºC, cells were harvested, washed, and resuspended in 700 μL sterile distilled water; strongly vortexed and 30 μL of 10 mg/mL lysostaphin (L-7386; Sigma) solution were added. The mixture was vortexed again and incubated in a water bath at 37°C for 18 h. A volume of 50 μL 20% sodium dodecyl sulphate (SDS; L-3771; Sigma) was added, and the mixture was boiled for 10 min and centrifuged at 13,000 × g for 15 min at 20°C. The supernatant was filtered through a 0.2 μm pore size membrane (Acrodisc 4192; Gelman). The protein assay was done according to Peterson modification of the Micro-Lowry method (P-5656; Sigma).^41^

Each protein extract was diluted to reach 1 μg/μL, and 20 μL of the solution was used for sodium dodecyl sulphate–polyacrylamide gel electrophoresis (SDS-PAGE), 10% separation gel and 4.5% stacking gel^42^ in a Protean II xi Cell (Bio-Rad), with a protein molecular weight marker of 205, 116, 97, 66, 45 and 29 kDa (SDS-6H; Sigma). Proteins were stained with 0.25% Coomassie Brilliant Blue (161-0400; Bio-Rad) or blotted onto nitrocellulose membranes according to Towbin et al.^43^ on a Trans-Blot Cell (Bio-Rad).

### Immunoblotting

Nitrocellulose membranes were blocked by placing the membrane in a 5% solution of non-fat dry milk for 1 h and washed with 0.05% Tween 20 (P-7949; Sigma) in phosphate-buffered saline (PBS-Tween).

The detection of specific IgG for *S. epidermidis* proteins was performed with blood serum, diluted 1:50, from four ewes with *S. epidermidis* IMI and two ewes without IMI, and milk whey, diluted 1:25, from five udder halves with *S. epidermidis* IMI from different ewes and two udder halves without IMI, also from different ewes. Specific IgA assessment was done in milk whey, diluted 1:25, from the same five udder halves with *S. epidermidis* IMI and the same two udder halves without IMI.

Membranes with the blots were incubated with serum and whey for 8 h at room temperature under gentle agitation, and then, the membranes were washed with PBS-Tween for 10 min. A negative control consisted of a membrane incubated with PBS.

For the detection of IgG, both in serum and whey, membranes were incubated for 14 h, at 4°C, under gentle agitation, with peroxidase-conjugated anti-ovine IgG (A-9452; Sigma) diluted to 1:5000. For IgA assessment in whey, after incubation at 4°C, during 14 h, with mouse anti-ovine IgA (MCA628; Serotec) diluted to 1:500, and washed, the membranes were incubated with peroxidase-conjugated rabbit anti-mouse IgG (61-6520; Zymed) diluted to 1:2000 for 8 h at room temperature under gentle agitation and then washed. For the detection of the signal, the peroxidase substrate 3,3′-diaminobenzidine (DAB, D-4293; Sigma) was used and blot images were analysed with Kodak 1D Digital Science (Eastman Kodak).

## Results

**Figure 1.**
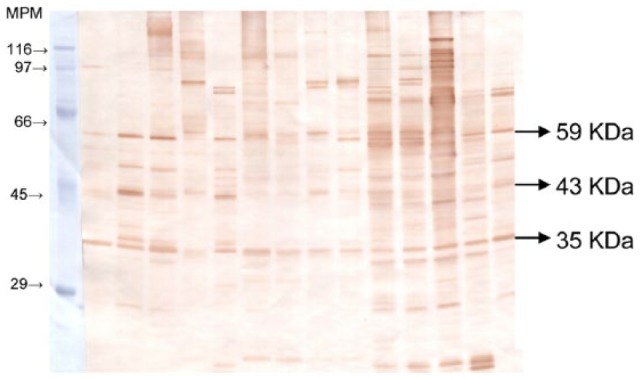
IgG detection in milk whey from a mastitic ewe. MPM: protein molecular weight marker (SDS-6H; Sigma).

A large variety of bacterial proteins was recognized by IgG from blood of both mastitic and healthy sheep and from milk of mastitic ewes and IgA from the milk of mastitic and healthy animals. Furthermore, some of these proteins appeared to be common to all isolates and were recognized by immunoglobulins of both blood and milk. Proteins with 59 and 43 kDa were recognized by all immunoglobulins screened in both blood and milk and in healthy and mastitic ewes. In milk, IgG and IgA were also able to recognize 35 kDa proteins (Figure 1).

**Figure 2.**
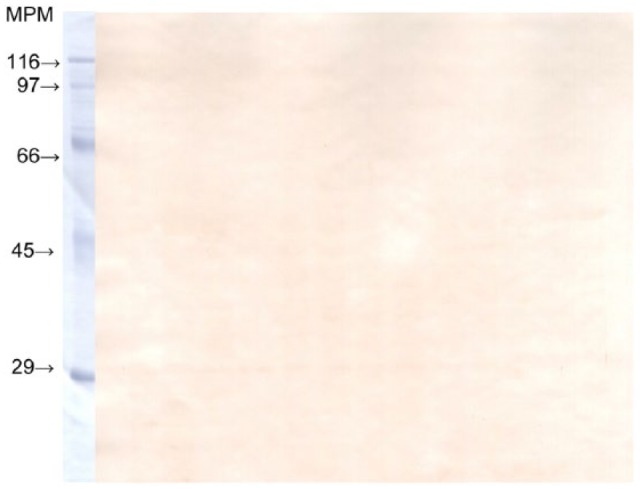
IgG detection in milk whey from a healthy ewe. MPM: protein molecular weight marker (SDS-6H; Sigma).

The protein profile recognized by circulating IgGs is relatively similar in both healthy and mastitic ewes. Likewise, IgA present in milk of both infected and control animals recognized analogous epitopes. Contrary to what was observed for IgG in blood and IgA in milk, there were no visible bands in membranes incubated with the healthy sheep’s whey for IgG, showing a similar result to the membrane incubated with PBS (Figure 2).

## Discussion

Results show that specific *S. epidermidis* IgG is present in the milk of ewes with *S. epidermidis* mastitis, but is not present in the milk of non-affected animals. Unlike other animal species, IgG represents the major immunoglobulin type in milk of sheep and other ruminants.^30^ In these animals, the substitution of IgA by IgG as the main immunoglobulin could be the result of an evolutionary adaptation to the lack of antibodies supplied through the placenta.^44^ Therefore, the main function of IgG in milk is to protect the offspring through passively transmitted maternal immunity, rather than the protection of the mammary gland. According to Berthon and Salmon,^29^ antibodies present in mammary secretions are specific for antigens and microorganisms of the mother’s digestive tract. Furthermore, Chang et al.^45^ mention that those antibodies are secreted mainly by intestinal-derived plasma cells.

The presence of specific IgG for staphylococcal proteins in the blood and not in milk of healthy sheep is probably due to the fact that the selective homing does not recognize those antigens as potential invaders of the mammary gland, which is a sterile environment.^46^ However, specific IgA is present in those animals.

In the blood of cattle and sheep, IgG1 is approximately 47% of all immunoglobulins and IgG2 represents around 37%, a relatively equivalent amount. However, in milk, IgG1 is close to 75% and IgG2 is merely around 5%.^47^ During an inflammatory process, IgG2 is carried out to milk by neutrophils,^48^ which bear specific receptors for the Fc fraction of IgG2.^30^ However, although there is an increase in passive transportation of serum proteins to milk after an inflammatory stimulation, the active transport of IgG1 is inhibited,^49^ and no active transport of IgG1 occurs in the completely involuted gland.^50^ This suggests that blood IgG1 has no major role in mammary gland infection, while IgG2 may be essential to fight invading microorganisms.

Rainard and Poutrel^51^ refer that the blood of adult ruminants contains opsonins for almost all mastitis causing bacteria. However, milk from a healthy mammary gland has a weak opsonizing activity, which has a tendency to increase during the inflammatory reaction. The local infusion of sheep mammary gland with microorganisms also induces an increase in milk opsonizing power. The fact that healthy ewes from our study only presented specific IgG in blood, but not in milk, where IgG1 represents the major isotype, may suggest that IgG2 constitutes the serum fraction specific for mastitis antigens. Others have suggested the relevance of IgG2 for mammary protection. Tomita et al.^33^ did not find IgG2 in the milk of non-vaccinated cows, but observed an increase in this isotype after vaccination and challenge. Subsequently to challenge, milk IgG2 increase was much higher in vaccinated animals compared with controls. Moreover, Prado et al.^36^ observed a superior IgG2 response, rather than IgG1, in the blood of cows subcutaneously vaccinated for mastitis.

**Figure 3.**
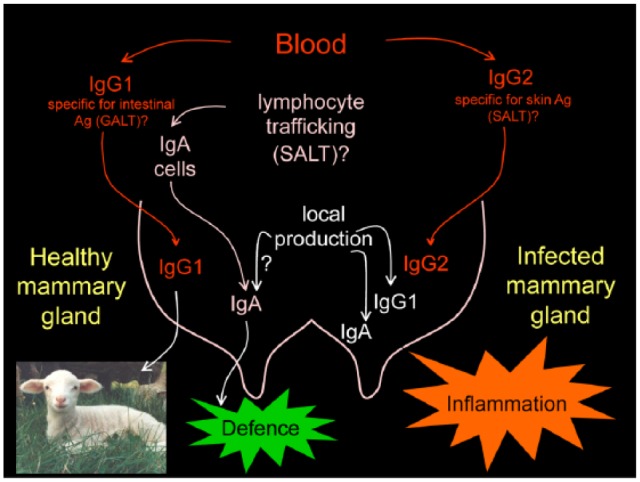
Proposed theory to explain immunoglobulin dynamics in the mammary gland. GALT: gut-associated lymphoid tissue; SALT: skin-associated lymphoid tissue.

Accordingly, the dynamics of IgG between blood and the mammary gland could be explained as follows: in blood, IgG1 is mainly specific for intestinal antigens. Most blood IgG1 is actively carried to the mammary gland during colostrum production and IgG2, specific for *S. epidermidis* proteins, is carried by the neutrophils exclusively during inflammation, when leucodiapdesis occurs and neutrophils accumulate in the mammary gland (Figure 3). This theory may explain the absence of specific IgG for mastitis antigens in the milk of healthy ewes.

The specificity difference between the two IgG subclasses may be related to the origin of the respective plasma cells that produce each isotype. It is currently accepted that the immune system of the ruminant mammary gland belongs to the skin-associated lymphoid tissue (SALT) rather than to the gut-associated lymphoid tissue (GALT), as it occurs in monogastric animals.^44^ Therefore, IgG1 should be essentially produced by plasma cells derived from stimulated B lymphocytes of the Peyer’s patches and specific for intestinal antigens. Instead, IgG2 should be secreted by SALT plasma cells, specific for skin antigens, where coagulase-negative *Staphylococci* predominate.

Subsequent to intramammary infection, beyond the flow of specific blood-derived IgG2, local production^52^ of specific IgA and IgG1 will further fight the invading agents. Local production of IgG1 and IgA was proven after local immunization, and the presence of substantial amounts of producing B lymphocytes of these two isotypes was detected in mammary tissue together with small amounts of IgG2 and IgM producing cells.^45^

Several authors refer that intramammary vaccination induces a better response than through other routes.^34,45^ Our results suggest that only local immunization will trigger a population of IgG-producing cells in the mammary gland.

The crucial value of IgA in the defence of the ewe mammary gland was also shown since specific IgA is present in the milk of healthy animals. Previous work on immune stimulation of the mammary gland of ruminants focused on the production of IgG2 and interferon gamma (IFN-γ), aiming at opsonization increase and phagocytosis improvement. Our results lead us to propose a stimulation strategy towards the increase in IgA. Leitner^53^ studies, in cattle, indicate that only local immunization will enhance specific IgA production in the mammary gland. Also, higher increase in IgA in the gland was obtained by others following local stimulation,^54^ contrasting with other administration routes.^55,56^

Besides local immunization, we suggest intradermic vaccination for sheep. If specific immunoglobulins for relevant antigens that target the mammary gland are produced by SALT B lymphocytes, this strategy is prone to induce an increase in specific IgG2. Camussone et al.^37^ did not get IgG2 in the whey of subcutaneously vaccinated cows in the supramammary lymph node area, although other authors have mentioned an IgG2 milk response.^33^ In their review on the efficacy of mastitis vaccines, Pereira et al.^22^ analysed 24 studies, from which only one referred the use of intradermal inoculation. Still, only serum antibodies were assessed and data on each IgG subclasses were not mentioned.^57^

As an exploratory study, the results are qualitative information, and interpretation of such type of information may be subject to bias. Accordingly, findings of exploratory research cannot be generalized to a wider population. The small sample size is a limitation of this study; however, the results are meaningful because the detection of IgG in whey was positive for all infected animals and negative for all healthy animals.

## Conclusion

Our results suggest that IgG2 is the serum IgG fraction specific for mastitis antigens. The detection of IgG subclasses for vaccine evaluation is vital to clarify this hypothesis.

Our work indicates that only local immunization will set off a population of IgG-producing cells in the mammary gland in response to mastitis antigens. Nevertheless, the use of an intradermic delivery of antigen might be indicated for mastitis prevention in sheep, considering that specific immunoglobulins for antigens relevant in the mammary gland are produced by SALT B lymphocytes. We believe that research on the immune outcome of intradermal vaccination for mastitis prevention should deserve further attention.

Finally, IgA seems to be a crucial asset for the defence of ewe mammary gland, and a stimulation strategy towards the increase in IgA should definitely be addressed for mastitis prevention to improve the immune exclusion of pathogens in the mammary gland.

## References

[1] Queiroga MC. Prevalence and aetiology of sheep mastitis in Alentejo region of Portugal. Small Rumin Res 2017; 153: 123–130.

[2] Bio C Bertollo F Bio R The subclinical mastitis in sheep duly slaughtered and milk quality. In: Proceedings of the 4th IDF international mastitis conference, Maastricht, 12–15 June, p. 919 Wageningen, Netherlands: Wageningen Academic Publishers.

[3] Bor A Winkler M Gootwine E. Non-clinical intramammary infection in lactating ewes and its association with clinical mastitis. Br Vet J 1989; 145(2): 178–184. 2713639 10.1016/0007-1935(89)90102-4

[4] Dario C Laudadio V Corsalini T et al Subclinical mastitis in sheep: occurrence, etiology and milk production in different genetic types. Agr Mediterr 1996; 126: 320–325.

[5] De la Cruz M Serrano E Montoro V et al Etiology and prevalence of subclinical mastitis in the Manchega sheep at mid-late lactation. Small Rumin Res 1994; 14(2): 175–180.

[6] González-Rodríguez MC Gonzalo C San Primitivo F et al Relationship between somatic cell count and intramammary infection of the half udder in dairy ewes. J Dairy Sci 1995; 78(12): 2753–2759. 8675758 10.3168/jds.s0022-0302(95)76906-5

[7] Jones JET Mastitis in sheep. In: Owen JB Axford RB (eds) Breeding for disease resistance in farm animals. Wallingford: CAB International, 1991, pp. 412–423.

[8] Las Heras A Fernández-Garayzábal JF Legaz E et al Importance of subclinical mastitis in milking sheep and diversity of aetiological agents. In: Barillet F Zervas NP (eds) Milking and milk production of dairy sheep and goats. Wageningen: EAAP, 1999, pp. 137–141.

[9] Marco JC Romeo M Esnal A et al Survey of intramammary infections in Latxa ewes. In: Proceedings of the sheep veterinary society, 1993, p. 228 Penicuik, Scotland: Sheep Veterinary Society.

[10] Pengov A. The role of coagulase-negative Staphylococcus spp. and associated somatic cell counts in the ovine mammary gland. J Dairy Sci; 2001; 84(3): 572–574. 11286408 10.3168/jds.S0022-0302(01)74509-2

[11] Romeo M Ziluaga I Marco JC. Diagnóstico in situ de la infección mamaria mediante palpación, California mastitis test y su seguimiento mediante recuento de células somáticas. Ovis 1998; 59: 61–77.

[12] Stefanakis A Boscos C Alexopoulos C et al Frequency of subclinical mastitis and observations on somatic cell counts in ewes’ milk in northern Greece. Anim Sci 1995; 61(1): 69–76.

[13] Vasileiou NGC Cripps PJ Ioannidi KS et al Extensive countrywide field investigation of subclinical mastitis in sheep in Greece. J Dairy Sci 2018; 101: 7297–7310. 29859691 10.3168/jds.2017-14075

[14] Watkins GH Burriel AR Jones JET A field investigation of subclinical mastitis in sheep in southern England. Br Vet J 1991; 147(5): 413–420. 1959012 10.1016/0007-1935(91)90083-Y

[15] Watson D Frankling N Davies H et al Survey of intramammary infections in ewes on the New England Tableland of New South Wales. Aust Vet J 1990; 67(1): 6–8. 2334377 10.1111/j.1751-0813.1990.tb07381.x

[16] Ziluaga DI Romeo M Marco JC. Prevalencia, patogenicidad y epidemiologia de los microorganismos implicados em procesos mamíticos del ganado ovino. Ovis 1998; 59: 27–49.

[17] Petridis IG Fthenakis GC. Administration of antibiotics to ewes at the beginning of the dry- period. J Dairy Res 2014; 81: 9–15. 24103579 10.1017/S0022029913000472

[18] Rajala-Schultz PJ Trres A Rajeev S et al Antimicrobial susceptibility of Staphylococcus spp. Isolated at Dry-off and Calving. In: Proceedings of the 4th IDF international mastitis conference, Maastricht, 12–15 June 2005, pp. 684–687. Wageningen, Netherlands: Wageningen Academic Publishers.

[19] Kaesbohrer A Schroeter A Tenhagen BA. Emerging antimicrobial resistance in commensal Escherichia coli with public health relevance. Zoonoses Public Health 2012; 59(Suppl. 2): 158–165. 22958260 10.1111/j.1863-2378.2011.01451.x

[20] Schukken YH Günther J Fitzpatrick J et al Host-response patterns of intramammary infections in dairy cows. Vet Immunol Immunopathol 2011; 144(3–4): 270–289. 21955443 10.1016/j.vetimm.2011.08.022

[21] Tiwari J Babra C Tiwari HK et al Trends in therapeutic and prevention strategies for management of bovine mastitis: an overview. J Vaccines Vaccin 2013; 4(2): 176.

[22] Pereira UP Oliveira DGS Mesquita LR et al Efficacy of *Staphylococcus aureus* vaccines for bovine mastitis: a systematic review. Vet Microbiol 2011; 148(2–4): 117–124. 21115309 10.1016/j.vetmic.2010.10.003

[23] Leitner G Lubashevsky E Glickman A et al Development of a *Staphylococcus aureus* vaccine against mastitis in dairy cows: I. Challenge trials. Vet Immunol Immunopathol 2003; 93(1–2): 31–38. 12753773 10.1016/s0165-2427(03)00051-5

[24] Leitner G Krifucks O Kiran MD et al Vaccine development for the prevention of staphylococcal mastitis in dairy cows. Vet Immunol Immunopathol 2011; 142(1–2): 25–35. 21524801 10.1016/j.vetimm.2011.03.023

[25] Paape MJ Shafer-Weaver K Capuco AV et al Immune surveillance of mammary tissue by phagocytic cells. Adv Exp Med Biol 2000; 480: 259–277. 10959434 10.1007/0-306-46832-8_31

[26] Oviedo-Boyso J Valdez-Alarcón JJ Cajero-Juárez M et al Innate immune response of bovine mammary gland to pathogenic bacteria responsible for mastitis. J Infect 2007; 54(4): 399–409. 16882453 10.1016/j.jinf.2006.06.010

[27] Rainard P Riollet C. Innate immunity of the Bovine mammary gland. Vet Res 2006; 37: 369–400. 16611554 10.1051/vetres:2006007

[28] Paape MJ Bannerman DD Zhao X et al The bovine neutrophil: structure and function in blood and milk Max. Vet Res 2003; 34: 597–627. 14556697 10.1051/vetres:2003024

[29] Berthon P Salmon H. Facteurs immunitaires des sécrétions mammaires. In: Martinet J Houdebine LM (eds) Biologie de la lactation. Nantes: Editions INSERM/INRA, 1993, pp. 389–414.

[30] Tizard IR. Veterinary immunology. 9th ed. Philadelphia, PA: Saunders, 2013.

[31] Burton JL Erskine RJ. Immunity and mastitis: some new ideas for an old disease. Vet Clin North Am Food Anim Pract 2003; 19(1): 1–45. 12682934 10.1016/s0749-0720(02)00073-7

[32] Alnakip ME Quintela-Baluja M Böhme K et al The immunology of mammary gland of dairy ruminants between healthy and inflammatory conditions. J Vet Med 2014; 2014: 659801. 26464939 10.1155/2014/659801 PMC4590879

[33] Tomita GM Nickerson SC Owens WE et al Influence of route of vaccine administration against experimental intramammary infection caused by Escherichia coli. J Dairy Sci 1998; 81(8): 2159–2164. 9749381 10.3168/jds.S0022-0302(98)75793-5

[34] Smith JL Hogan JS Smith KL. Efficacy of intramammary immunization with an Escherichia coli J5 bacterin. J Dairy Sci 1999; 82(12): 2582–2588. 10629804 10.3168/jds.S0022-0302(99)75513-X

[35] Prenafeta A March R Foix A et al Study of the humoral immunological response after vaccination with a *Staphylococcus aureus* biofilm-embedded bacterin in dairy cows: possible role of the exopolysaccharide specific antibody production in the protection from *Staphylococcus aureus* induced mastitis. Vet Immunol Immunopathol 2010; 134(3–4): 208–217. 19836084 10.1016/j.vetimm.2009.09.020

[36] Prado ME Almeida RA Ozen C et al Vaccination of dairy cows with recombinant *Streptococcus uberis* adhesion molecule induces antibodies that reduce adherence to and internalization of *S. uberis* into bovine mammary epithelial cells. Vet Immunol Immunopathol 2011; 141(3–4): 201–208. 21477869 10.1016/j.vetimm.2011.02.023

[37] Camussone CM Veaute CM Pujato N et al Immune response of heifers against a *Staphylococcus aureus* CP5 whole cell and lysate vaccine formulated with ISCOM Matrix adjuvant. Res Vet Sci 2014; 96(1): 86–94. 24210331 10.1016/j.rvsc.2013.10.004

[38] Piepers S Prenafeta A Verbeke J et al Immune response after an experimental intramammary challenge with killed *Staphylococcus aureus* in cows and heifers vaccinated and not vaccinated with Startvac, a polyvalent mastitis vaccine. J Dairy Sci 2016; 100(1): 769–782. 27816241 10.3168/jds.2016-11269

[39] National Mastitis Council (US). Microbiological procedures for the diagnosis of bovine udder infection and determination of milk quality. 4th ed. Verona: NMC, 2004, p. 47.

[40] Pereira SFF Couto I Queiroga C et al Molecular identification by ITS-PCR of staphylococcal isolates from ovine sub-clinical mastitis. In: Abstracts of 1st FEMS congress of European microbiologists, Ljubljana, 29 June–3 July, paper no. P7–24, p. 280 Delft: FEMS.

[41] Peterson GL A simplification of the protein assay method of Lowry et al. which is more generally applicable. Anal Biochem 1977; 83(2): 346–56. 603028 10.1016/0003-2697(77)90043-4

[42] Laemmli UK. Cleavage of structural proteins during the assembly of the head of bacteriophage T4. Nature 1970; 227: 680–685. 5432063 10.1038/227680a0

[43] Towbin H Staehelin T Gordon J. Electrophoretic transfer of proteins from polyacrylamide gels to nitrocellulose sheets: procedure and some applications. Proc Natl Acad Sci U S A 1979; 76(9): 4350–4354. 388439 10.1073/pnas.76.9.4350 PMC411572

[44] Kehrli ME Harp JA. Immunity in the mammary gland. Vet Clin North Am Food Anim Pract 2001; 17(3): 495–516. 11692505 10.1016/s0749-0720(15)30003-7

[45] Chang CC Winter AJ Norcross NL. Immune response in the bovine mammary gland after intestinal, local, and systemic immunization. Infect Immun 1981; 31(2): 650–659. 7012016 10.1128/iai.31.2.650-659.1981 PMC351359

[46] Rainard P. Mammary microbiota of dairy ruminants: fact or fiction? Vet Res 2017; 48(1): 25. 28412972 10.1186/s13567-017-0429-2 PMC5392980

[47] Butler JE. Immunoglobulin diversity, B-cell and antibody repertoire development in large farm animals. Rev Sci Tech 1998; 17(1): 43–70. 9638800 10.20506/rst.17.1.1096

[48] Sordillo LM Shafer-Weaver K DeRosa D. Immunobiology of the mammary gland. J Dairy Sci 1997; 80(8): 1851–1865. 9276826 10.3168/jds.S0022-0302(97)76121-6

[49] Lascelles AK. The immune system of the ruminant mammary gland and its role in the control of mastitis. J Dairy Sci 1979; 62(1): 154–160. 379059 10.3168/jds.s0022-0302(79)83216-6

[50] Colditz IG Watson DL. The immunophysiological basis for vaccinating ruminants against mastitis. Aust Vet J 1985; 62(5): 145–153. 3899085 10.1111/j.1751-0813.1985.tb07276.x

[51] Rainard P Poutrel B. Protection immunitaire de la glande mammaire. In: Martinet J Houdebine LM (eds) Biologie de la lactation. Nantes: INSERM/INRA, 1993, pp. 415–429.

[52] Fragkou IA Mavrogianni VS Papaioannou N et al Presence of sub-epithelial lymphoid tissues in the teat of ewe-lambs and adult ewes. Small Rumin Res 2007; 70(2–3): 286–291.

[53] Leitner G The mammary gland defense system and mucosal immunity. In: Proceedings of the 4th IDF international mastitis conference, Maastricht, 12–15 June 2005, p. 760 Wageningen Academic Publishers.

[54] Hodgkinson AJ Carpenter EA Smith CS et al Effects on adhesion molecule expression and lymphocytes in the bovine mammary gland following intra-mammary immunisation. Vet Immunol Immunopathol 2009; 131(1–2): 110–116. 19376595 10.1016/j.vetimm.2009.03.016

[55] Pellegrino M Giraudo J Raspanti C et al Efficacy of immunization against bovine mastitis using a *Staphylococcus aureus* avirulent mutant vaccine. Vaccine 2010; 28(28): 4523–4528. 20450870 10.1016/j.vaccine.2010.04.056

[56] Middleton JR Luby CD Adams DS. Efficacy of vaccination against staphylococcal mastitis: a review and new data. Vet Microbiol 2009; 134(1–2): 192–198. 19010613 10.1016/j.vetmic.2008.09.053

[57] Carter EW Kerr DE. Optimization of DNA-based vaccination in cows using green fluorescent protein and protein A as a prelude to immunization against staphylococcal mastitis. J Dairy Sci 2003; 86(4): 1177–1186. 12741542 10.3168/jds.S0022-0302(03)73701-1

